# Muscle Activity in Pre-Treatment and Post-Treatment Oral Submucous Fibrosis Patients: Electromyography Study

**DOI:** 10.1155/2022/2826862

**Published:** 2022-09-30

**Authors:** Santosh R Patil, G. Maragathavalli, D. N. S. V. Ramesh, Mohammad Khursheed Alam

**Affiliations:** ^1^Department of Oral Medicine and Radiology, Saveetha Dental College and Hospitals, Chennai, India; ^2^Department of Oral Medicine and Radiology, A. M. E'S Dental College and Hospital, Raichur, India; ^3^Department of Preventive Dentistry, College of Dentistry, Jouf University, Sakakah, Saudi Arabia; ^4^Department of Public Health, Faculty of Allied Health Sciences, Daffodil International University, Dhaka 1207, Bangladesh

## Abstract

**Background:**

Oral submucous fibrosis (OSMF) is a premalignant condition of insidious onset which affects the oral mucosa, pharynx, and esophagus. The muscles of mastication are known to be affected resulting in limited mouth opening. Electromyography (EMG) is a sophisticated method of measuring and evaluating muscle activity. Previously, EMG was primarily utilized in medical sciences, but it is currently being used extensively in both the medical and dentistry fields.

**Objectives:**

The aim of the study is to evaluate the electromyographic activity of masseter muscle in OSMF patients before and after treatment and to compare with healthy controls.

**Materials and Methods:**

This prospective case-control clinical study comprised 180 OSMF patients who were divided into four groups and 45 healthy individuals served as the control group. The OSMF individuals were injected with hyaluronidase 1,500 IU mixed in 1.5 ml of dexamethasone and 0.5 ml of lignocaine HCL intralesionally twice a week for one month along with a basic physiotherapy regimen consisting of mouth exercises two times daily. The control subjects were given placebo capsules. The treatment was carried out for a month and the electromyographic masseter muscle activity was evaluated among the OSMF patients and control group before and after treatment.

**Results:**

The results revealed that the electromyographic activity of master muscles in OSMF patients showed increased activity when compared with healthy controls. Patients with OSMF showed decreased muscle activity after treatment.

**Conclusion:**

When compared with healthy controls, OSMF patients had higher electromyographic activity of the masseter muscles and the muscle activity was decreased following treatment. In OSMF patients, EMG may help in determining the involvement of the mastication and facial expression muscles. It can also be used as a diagnostic tool to assess the treatment outcome of muscle activity in OSMF patients.

## 1. Introduction

OSMF is a long-standing enfeebling, premalignant condition that involves the oral mucosa, pharyngeal mucosa, and upper digestive tract, portrayed by limited mouth opening because of inflammatory changes and underlying fibrotic changes of the submucosal tissues. [1] Masticatory muscle involvement and replacement with fibrous tissue were noted in individuals with OSMF. The exact extent of fibrosis and its role in causing trismus are determined by several factors including the anatomical and physiological integrity of the underlying musculature. [2].

Muscle atrophy, degeneration, and necrosis were found in considerable amounts in OSMF subjects in prior studies. [3–5] These muscle alterations can occur as a result of the underlying disease process, with atrophy occurring as a result of the decreased muscular function caused by fibrosis, or as a crucial component of the disease process. [3, 4] Muscle degeneration has also been confirmed in OSMF patients in previous histopathology and electron microscopy studies. [2, 5].

There is a direct association between areca chewing habit and the development of OSMF; the reason being exaggerated forces on the masseter muscle due to vigorous chewing for prolonged periods. The masseter is a strong elevator muscle of the mandible. It covers more surface area of the mandible and remains close to the muscles of facial expression; these two factors are responsible for the early involvement of the masseter muscle in OSMF. [6].

Electromyography (EMG) is a research technique that involves the generation, recording, and analysis of myoelectric signals. EMG measures the electrical potential generated by muscle cells when they are electrically or neurologically engaged. Medical and dental issues can be detected by analyzing the signals. The motor unit is the structural foundation of electromyography. The previous study by Kant et al. evaluated the masticatory muscle activity in OSMF patients but the study was carried out with a smaller sample size and no therapeutic intervention was carried out [6]. To further evaluate the observations, the present study is carried out with a larger sample size, and the activity of masseter muscle in terms of duration and amplitude was evaluated before and after treatment in OSMF patients.

The hypothesis to be tested as there will be a significant difference in masticatory muscle activity before and after treatment with intralesional corticosteroids.

## 2. Materials and Methods

### 2.1. Study Design

It was a prospective, case-control study carried out after obtaining clearance from the institutional ethical committee (Reference# SDC/Ph.D/07/18/44).

### 2.2. Setting

The present study was carried out in AMES Dental College and Hospital, India, for a duration of three years, from January 2019 to December 2021.

### 2.3. Participants

The study group comprised 180 clinically diagnosed OSMF patients and the control group included 45 healthy individuals. Patients with, infections and inflammatory causes responsible for trismus, intraarticular causes of trismus, myofascial pain dysfunction syndrome, trauma to the maxillofacial skeleton, dermatomyositis and myositis ossificans, temporomandibular joint arthritis, and arthralgia, scleroderma, systemic disease, gingivitis and periodontitis, parafunctional habits, previous history of treatment for OSMF were excluded.

### 2.4. Measurements

Detailed case history including personal history was obtained from all the participants. The clinical examination was carried out for all patients and recorded in a proforma. The demographic data along with habit details were recorded before the clinical examination.

The mouth opening of all patients and controls was recorded using a Vernier caliper, to measure the distance between the upper and lower central incisor edges at the maximum unaided mouth opening. Patients were divided into four groups based on inter-incisal mouth opening according to Lai et. al [7].

- Group A (mouth opening more than 35 mm)
- Group B (mouth opening between 30 and 35 mm)
- Group C (mouth opening between 20 and 30 mm)
- Group D (mouth opening less than 20 mm)

The electromyographic activity of the study participants was recorded using NeuroStim NS2, a Window based computerized EMG/NCV/EP System.

The participants were instructed to sit up straight and with their heads in a natural position. They were also told not to consume betel nut in any form for two hours prior to electromyography. The skin was next cleansed with a cotton swab soaked in 70% alcohol. A small amount of electromyographic gel was used to position the electrodes on the recording location and the electrodes. Collodion adhesive was used to hold two silver-silver chloride surface electrodes (active electrode and reference electrode) in place. The electrode pair was spaced at a spacing of 3-4 mm between them. For electromyography recording, the paired electrodes serve as exploring electrodes. On the lateral side of the neck, one ground electrode was placed. Microvolts were used to measure muscle activation and muscle activity was recorded in microvolts. The activity of the master muscle was recorded in the relaxed state and maximum voluntary contracted state.

**Figure 1:**
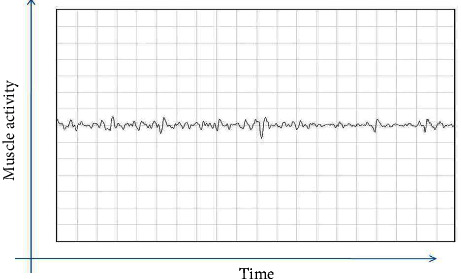
Electromyography recording of a healthy individual before treatment.

**Figure 2:**
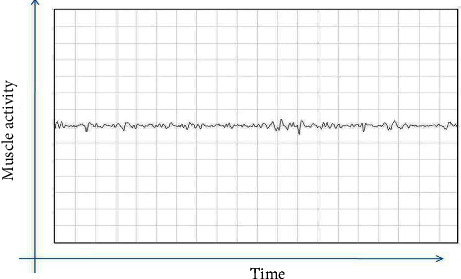
Electromyography recording of a healthy individual after treatment.

**Figure 3:**
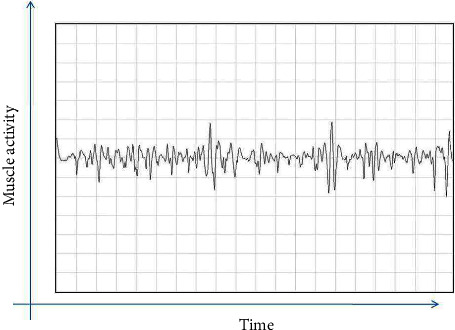
Electromyography recording of OSMF patients before treatment.

**Figure 4:**
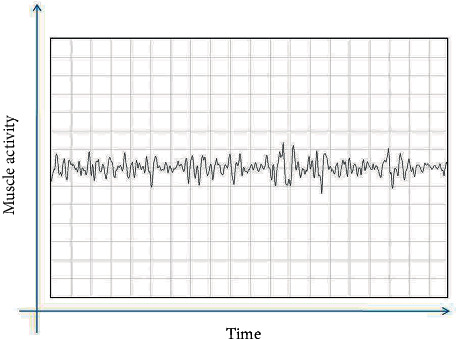
Electromyography recording of OSMF patients after treatment.

Exploring electrodes for recording electromyography were oriented parallel to the direction of the masseter muscle. The site of recording activity was in the thickest part of the masseter muscle, close to the level of the occlusal plane, approximately in the middle of the mediolateral distance of the ramus. The activity of the masseter muscle was first recorded in a relaxed state and then in a maximum voluntary contracted state, by asking the patient to clench his teeth. The same was repeated for the left masseter muscle (Figures 1–4).

After initial recording of EMG, all the OSMF patients were injected with hyaluronidase 1,500 IU mixed in 1.5 ml of dexamethasone and 0.5 ml of lignocaine HCL intralesionally twice a week for one month along with a basic physiotherapy regimen consisting of mouth exercises two times daily. The control subjects were given placebo capsules. The treatment was carried out for a month and the muscle activity of all participants was revaluated after the completion of the treatment. The OSMF subjects were informed about the disease and its precancerous potential and were encouraged to stop using the areca nut and tobacco.

### 2.5. Statistical Methods

Statistical analysis was done using Statistical Package for Social Sciences (SPSS), IBM Statistics version 22.0 statistical analysis software. The values were represented in number, mean and standard deviation (SD). Significance is assessed at a 5% level of significance. The normality of the data was assessed using the Kolmogorov–Smirnov test. Student's *t*-test, paired *t*-test, one-way analysis of variance (ANOVA), and Tukey Post Hoc test was performed.

## 3. Results

In the present study, there were 43 males and 2 females each in groups A and B, 44 males and 1 female each in groups C and D, and 39 males and 6 females in the control group. The mean age was 30.14 ± 5.62 years and 32.27 ± 2.16 years among cases and controls, respectively.

Masseter muscle duration before and after treatment (Tables 1–4).

We noted a gradual increase in the duration of masseter muscle from group A to group C (35 mm to 20 mm of mouth opening). Tukey's post hoc multiple comparisons show a highly significant difference between group D (<20 mm of mouth opening) and other groups both before and after treatment. Although there was a difference in duration between other groups that difference was not significant (*p* > 0.05).

Masseter muscle amplitude before and after treatment (Tables 5–8).

We noted a gradual increase in the amplitude of masseter muscle from group A to group C (35 mm to 20 mm of mouth opening). Tukey's post hoc multiple comparisons show a highly significant difference between group D and other groups both before and after treatment. Although there was a difference in amplitude between other groups that difference was not significant (*p* > 0.05).

### 3.1. Sidewise Comparison of Masseter Muscle Duration and Amplitude

No significant difference was observed between the right and left side masseter muscle duration and amplitude in any of the groups (Tables 9 and 10).

## 4. Discussion

In the present study, the activity of the left and right master muscles in 180 patients with signs and symptoms of OSMF and 45 control subjects was recorded using electromyography.

The recording and study of the electrical potentials of muscles are known as electromyography. Man has always been interested in learning more about himself and his surroundings. This led to several ground-breaking technologies that made life easier for humans. In the realm of bioelectricity, much study has been done and documented, leading to today's electrodiagnostic methods. The use of EMG as a diagnostic tool has been a benefit to medicine. It has a significant impact on several parts of clinical medicine and dentistry. The use of electromyography in the research of functional jaw muscle morphology has a long history. [8] Normal and healthy jaw muscles can both contract and release with sufficient power and coordination during mandibular rest. When making a diagnosis of muscle dysfunction; however, palpation and visual inspection of these basic processes are not usually sufficient. Electromyography is the sole reliable approach for objectively documenting a patient's muscle function when a more thorough understanding is required. Both indwelling electrodes and surface recordings have been used to study the electromyographic activity of masticatory muscles in clinical practice and research. [9, 10].

EMG is frequently used in clinical and research settings. EMG is more commonly used in dentistry for temporomandibular joint (TMJ) disorder, TMJ dysfunction, dystonia, head and neck muscular disorders, cranial nerve lesions, and seizure disorders. EMG is also used to diagnose other disorders that are related to muscle tissue and nerve degeneration, such as amyotrophic lateral sclerosis (ALS) and myasthenia gravis (MG). Furthermore, EMG is useful in the identification of facial muscles during orthodontic treatment, particularly regarding the neuromuscular approach and face discomfort caused using functional appliances. The EMG equipment is capable of examining various essential muscles involved in eating, swallowing, and head position (typically masseter, temporalis, anterior and posterior digastric, and sternocleidomastoid). [10].

Electromyographic activity was recorded in rest and maximum voluntary contracted state. During the rest position, the muscles were electrically silent in groups A, B, C, and D and the control group.

There was an increase in the duration and amplitude of masseter muscle from group A to group C (35 mm to 20 mm of mouth opening). We did not notice any significant difference in masseter muscle duration and amplitude within all groups and between groups before and after treatment. Tukey's post hoc multiple comparisons showed a highly significant difference between group D and other groups both before and after treatment. Although there was a difference in duration and amplitude between other groups that difference was not significant. Upon sidewise comparison, no significant difference was noted with respect to masseter muscle duration and amplitude.

Ferrario et al. [11] reported 181.9 *µ*V mean maximum voluntary clench potential for males and 156.8 *µ*V for females. The authors noted that at rest, no gender differences were found; in both sexes, temporal muscle potentials were higher than masseter muscle. Geogiakaki et al. [12] observed 379.0 ± 56.0 *µ*V mean muscle activity for the right muscle and 372.3 ± 73.2 *µ*V for the left muscle. The amplitude of the masseter muscle on the right (359.07 ± 20.41 *µ*V) and left (359.99 ± 17.14 *µ*V) side before treatment in the control group of the present study is not in agreement with these findings. This might be due to the difference in the methodology adopted in those studies.

The contracted state of the muscle was investigated because more motor units are recruited in this condition, which is highly dependent on muscular force production, such as fiber length and velocity. A complete recruitment pattern is created during maximal voluntary contraction, which is referred to as an interference pattern of EMG amplitude. [13].

There was a decrease in the mean activity of the muscles after the treatment. This might be related to the patient's treatment regimen, which included advising them to quit the tobacco and areca nut chewing habits, and administering intralesional injections of dexamethasone, hyaluronidase, and mouth opening exercises.

It has been observed that discontinuation of habit reduces masticatory stressors, which lowers muscular strength and resilience to fatigue, resulting in a reduction in muscle thickness and activity [14]. Corticosteroids are known to reduce inflammatory responses, avoiding fibrosis by lowering fibroblastic proliferation, sub-regulating collagen synthesis, and downregulating collagenase production. Hyaluronidase works by breaking down hyaluronic acid, reducing or decreasing inflammatory responses, and limiting the function of sensitized lymphocytes after activation by certain antigens [15]. Dexamethasone and hyaluronidase are hypothesized to be responsible for avoiding trismus and the production of fibrous bands [16]. As a result, it aids in the reduction of masticatory tension and resistance to function. [17].

When compared to before treatment, posttreatment electromyographic activity in OSMF patients was lower in this study. This finding was comparable with the findings of Sinha et al.; the authors observed an increase in electromyographic activity of the masseter muscle in OSMF patients and a corresponding decrease in muscle activity after therapy with corticosteroids administered intralesionally. [18].

One of the concerning factors in OSMF patients is the poor oral hygiene and periodontal tissue destruction. Studies in the literature reported that OSMF patients have poorer oral hygiene and increased gingival bleeding. [19, 20] Chatrchaiwiwatana suggested that the areca nut decreases the resistance to local factors and causes increased calculus deposition. [21] The reason for this could be decreased mouth opening which limits the oral hygiene practice methods like brushing and flossing among OSMF patients.

### 4.1. Limitations and Future Perspectives

Our study has a limitation in that surface electrodes were used for activity recording, and their accuracy in capturing the action potential of the muscle under study may be affected when action potentials from nearby muscle fibers are mixed together. In contrast, needle electrodes, which are inserted directly into the target muscle, are more effective at capturing the action potential of the desired muscle. Further studies using needle electrodes are suggested to overcome this drawback.

## 5. Conclusion

When compared with healthy controls, OSMF patients had a higher electromyographic activity of the masseter muscles and the muscle activity was decreased following treatment.

Electromyography, which involves finding electrical potentials in muscles, is the most objective and reliable tool for imaging muscle activity and efficiency. EMG has a variety of applications in general dentistry, including observation, diagnostic, and therapeutic purposes. In individuals with OSMF, EMG can help determine the involvement of the mastication and facial expression muscles. It can also be used as a diagnostic tool to assess the treatment outcome of muscle activity in OSMF patients.

## Figures and Tables

**Table 1:** Right side masseter muscle duration before and after treatment.

Groups	*Before*		*After*		Mean difference	% of mean change	*t* value	*P* value
	Mean	SD	Mean	SD				
Group A	7.26	0.36	7.25	0.31	0.01	0.14	0.277	0.783
Group B	7.36	0.39	7.26	0.49	0.10	1.36	1.490	0.143
Group C	7.38	0.62	7.30	0.56	0.08	1.08	1.735	0.090
Group D	5.23	0.51	5.13	0.56	0.10	1.91	1.463	0.151
Control	7.24	0.18	7.21	0.19	0.03	0.41	0.942	0.351
F value	204.328	204.505	—					
*p* value	0.000^*∗*^	0.000^*∗*^						

*∗p* < 0.05, Group A (mouth opening more than 35 mm), Group B (mouth opening between 30 and 35 mm), Group C (mouth opening between 20 and 30 mm), and Group D (mouth opening less than 20 mm).

**Table 2:** Multiple comparison by Tukey Post Hoc test.

Comparisons	*Before*		*After*	
	Mean difference	*P* value	Mean difference	*P* value
Group A vs Group B	0.10	0.813	0.01	1.000
Group A vs Group C	0.12	0.686	0.05	0.985
Group A vs Group D	2.03	0.000^*∗*^	2.12	0.000^*∗*^
Group A vs control	0.02	1.000	0.04	0.997
Group B vs Group C	0.02	0.999	0.04	0.994
Group B vs Group D	2.13	0.000^*∗*^	2.13	0.000^*∗*^
Group B vs control	0.12	0.707	0.05	0.990
Group C vs Group D	2.15	0.000^*∗*^	2.17	0.000^*∗*^
Group C vs control	0.14	0.565	0.09	0.905
Group D vs control	2.01	0.000^*∗*^	2.08	0.000^*∗*^

*∗p* < 0.05, same as previous comment.

**Table 3:** Left side masseter muscle duration before and after treatment.

Groups	*Before*		*After*		Mean difference	% of mean change	*t* value	*P* value
	Mean	SD	Mean	SD				
Group A	7.40	0.45	7.36	0.39	0.04	0.54	0.876	0.386
Group B	7.44	0.51	7.40	0.44	0.04	0.54	1.564	0.125
Group C	7.50	0.64	7.43	0.55	0.07	0.93	1.833	0.074
Group D	5.17	0.14	5.12	0.21	0.05	0.97	1.306	0.198
Control	7.25	0.29	7.21	0.26	0.04	0.55	1.137	0.262
F value	232.132	290.935	—					
*p* value	0.000^*∗*^	0.000^*∗*^						

*∗p* < 0.05, Group A (mouth opening more than 35 mm), Group B (mouth opening between 30 and 35 mm), Group C (mouth opening between 20 and 30 mm), and Group D (mouth opening less than 20 mm).

**Table 4:** Multiple comparison by Tukey Post Hoc test.

Comparisons	*Before*		*After*	
	Mean difference	*P* value	Mean difference	*P* value
Group A Vs Group B	0.04	0.987	0.04	0.994
Group A Vs Group C	0.10	0.778	0.07	0.936
Group A Vs Group D	2.23	0.000^*∗*^	2.24	0.000^*∗*^
Group A Vs control	0.15	0.513	0.15	0.361
Group B Vs Group C	0.06	0.967	0.03	0.996
Group B Vs Group D	2.27	0.000^*∗*^	2.28	0.000^*∗*^
Group B Vs control	0.19	0.231	0.19	0.173
Group C Vs Group D	2.33	0.000^*∗*^	2.31	0.000^*∗*^
Group C Vs control	0.25	0.053	0.22	0.073
Group D Vs control	2.08	0.000^*∗*^	2.09	0.000^*∗*^

*∗p* < 0.05, Group A (mouth opening more than 35 mm), Group B (mouth opening between 30 and 35 mm), Group C (mouth opening between 20 and 30 mm), and Group D (mouth opening less than 20 mm.

**Table 5:** Right side masseter muscle amplitude before and after treatment.

Groups	*Before*		*After*		Mean difference	% of mean change	*t* value	*P* value
	Mean	SD	Mean	SD				
Group A	361.08	26.13	360.48	24.73	0.60	0.17	0.285	0.777
Group B	362.37	16.92	362.14	20.03	0.23	0.06	0.146	0.885
Group C	363.15	16.51	362.99	16.52	0.16	0.04	0.078	0.938
Group D	346.23	23.10	345.83	30.06	0.40	0.12	0.172	0.864
Control	359.07	20.41	358.41	14.04	0.66	0.18	0.311	0.757
F value	4.983	4.628	—					
*p* value	0.001^*∗*^	0.001^*∗*^						

*∗p* < 0.05, Group A (mouth opening more than 35 mm), Group B (mouth opening between 30 and 35 mm), Group C (mouth opening between 20 and 30 mm), and Group D (mouth opening less than 20 mm).

**Table 6:** Multiple comparison by Tukey Post Hoc test.

Comparisons	*Before*		*After*	
	Mean difference	*P* value	Mean difference	*P* value
Group A Vs Group B	1.29	0.998	1.66	0.996
Group A Vs Group C	2.07	0.990	2.51	0.982
Group A Vs Group D	14.85	0.008^*∗*^	14.65	0.014^*∗*^
Group A Vs control	2.01	0.991	2.07	0.992
Group B Vs Group C	0.78	1.000	0.85	1.000
Group B Vs Group D	16.14	0.003^*∗*^	16.31	0.004^*∗*^
Group B Vs control	3.30	0.945	3.73	0.928
Group C Vs Group D	16.92	0.002^*∗*^	17.16	0.002^*∗*^
Group C Vs control	4.08	0.887	4.58	0.858
Group D Vs control	12.84	0.032^*∗*^	12.58	0.049^*∗*^

*∗p* < 0.05, Group A (mouth opening more than 35 mm), Group B (mouth opening between 30 and 35 mm), Group C (mouth opening between 20 and 30 mm), and Group D (mouth opening less than 20 mm).

**Table 7:** Left side masseter muscle amplitude before and after treatment.

Groups	*Before*		*After*		Mean difference	% Of mean change	*t* value	*P* value
	Mean	SD	Mean	SD				
Group A	361.57	33.62	361.49	39.38	0.08	0.02	0.030	0.976
Group B	361.82	16.84	361.62	17.88	0.20	0.06	0.096	0.924
Group C	362.44	21.85	362.34	29.02	0.10	0.03	0.027	0.978
Group D	344.97	22.23	343.46	26.21	1.51	0.44	1.318	0.194
Control	359.99	17.14	359.84	21.51	0.15	0.04	0.034	0.973
F-value	4.635	3.766	—					
*p*-value	0.001^*∗*^	0.006^*∗*^						

*∗p* < 0.05, Group A (mouth opening more than 35 mm), Group B (mouth opening between 30 and 35 mm), Group C (mouth opening between 20 and 30 mm), and Group D (mouth opening less than 20 mm).

**Table 8:** Multiple comparison by Tukey Post Hoc test.

Comparisons	*Before*		*After*	
	Mean difference	*P* value	Mean difference	*P* value
Group A Vs Group B	0.25	1.000	0.13	1.000
Group A Vs Group C	0.87	1.000	0.85	1.000
Group A Vs Group D	16.60	0.007^*∗*^	18.03	0.020^*∗*^
Group A Vs control	1.58	0.998	1.65	0.999
Group B Vs Group C	0.62	1.000	0.72	1.000
Group B Vs Group D	16.85	0.006^*∗*^	18.16	0.018^*∗*^
Group B Vs control	1.83	0.996	1.78	0.998
Group C Vs Group D	17.47	0.004^*∗*^	18.88	0.013^*∗*^
Group C Vs control	2.45	0.987	2.50	0.993
Group D Vs control	15.02	0.020^*∗*^	16.38	0.044^*∗*^

*∗p* < 0.05, Group A (mouth opening more than 35 mm), Group B (mouth opening between 30 and 35 mm), Group C (mouth opening between 20 and 30 mm), and Group D (mouth opening less than 20 mm).

**Table 9:** Sidewise comparison of masseter muscle duration.

Groups	Times	*Right*		*Left*		Mean difference	*t* value	*p* value
		Mean	SD	Mean	SD			
*Group A*	Before	7.26	0.36	7.40	0.45	0.14	1.628	0.107
	After	7.25	0.31	7.36	0.39	0.11	1.576	0.119

*Group B*	Before	7.36	0.39	7.44	0.51	0.08	0.900	0.370
	After	7.26	0.49	7.40	0.44	0.14	1.421	0.159

*Group C*	Before	7.38	0.62	7.50	0.64	0.12	0.956	0.342
	After	7.30	0.56	7.43	0.55	0.13	1.135	0.259

*Group D*	Before	5.23	0.51	5.17	0.14	0.06	0.864	0.390
	After	5.13	0.56	5.12	0.21	0.01	0.094	0.925

*Control*	Before	7.24	0.18	7.25	0.29	0.01	0.198	0.843
	After	7.21	0.19	7.21	0.26	0.00	0.019	0.985

*∗p* < 0.05, Group A (mouth opening more than 35 mm), Group B (mouth opening between 30 and 35 mm), Group C (mouth opening between 20 and 30 mm), and Group D (mouth opening less than 20 mm).

**Table 10:** Sidewise comparison of masseter muscle amplitude.

Groups	Times	*Right*		*Left*		Mean difference	*t* value	*p* value
		Mean	SD	Mean	SD			
*Group A*	Before	361.08	26.13	361.57	33.62	0.49	0.077	0.939
	After	360.48	24.73	361.49	39.38	1.01	0.147	0.883

*Group B*	Before	362.37	16.92	361.82	16.84	0.55	0.154	0.878
	After	362.14	20.03	361.62	17.88	0.52	0.128	0.899

*Group C*	Before	363.15	16.51	362.44	21.85	0.71	0.174	0.862
	After	362.99	16.52	362.34	29.02	0.65	0.131	0.896

*Group D*	Before	346.23	23.10	344.97	22.23	1.26	0.263	0.793
	After	345.83	30.06	343.46	26.21	2.37	0.399	0.691

*Control*	Before	359.07	20.41	359.99	17.14	0.92	0.232	0.817
	After	358.41	14.04	359.84	21.51	1.43	0.374	0.709

*∗p* < 0.05, Group A (mouth opening more than 35 mm), Group B (mouth opening between 30 and 35 mm), Group C (mouth opening between 20 and 30 mm), and Group D (mouth opening less than 20 mm).

## Data Availability

The data that support the findings of this study are available from the corresponding author upon reasonable request.
